# Prevalence and predictors of eating disorders: A cross-sectional survey of medical students at King Abdul-Aziz University, Jeddah

**DOI:** 10.12669/pjms.38.6.5033

**Published:** 2022

**Authors:** Ranya A Ghamri, Asma M Alahmari, Lama S Alghamdi, Sarah F Alamoudi, Mada M Barashid

**Affiliations:** 1 Ranya A Ghamri, Department of Family Medicine, Faculty of Medicine, King Abdulaziz University, Jeddah, Saudi Arabia; 2 Asma M Alahmari, Medical Intern, Faculty of Medicine, King Abdulaziz University, Jeddah, Saudi Arabia; 3 Lama S Alghamdi, Medical Intern, Faculty of Medicine, King Abdulaziz University, Jeddah, Saudi Arabia; 4 Sarah F Alamoudi, Medical Intern, Faculty of Medicine, King Abdulaziz University, Jeddah, Saudi Arabia; 5 Mada M Barashid, Medical Intern, Faculty of Medicine, King Abdulaziz University, Jeddah, Saudi Arabia

**Keywords:** Eating disorder, Dietary habits, Medical students, Eating behaviors, Saudi Arabia

## Abstract

**Objectives:**

Eating disorders, such as anorexia nervosa and bulimia nervosa are psychiatric and public health issues that are prevalent among medical students, especially females. This study’s objective was to examine the epidemiology of eating disorders, specifically their associated risk factors, such as body mass index (BMI), gender, academic year of studies, and socio-demographic factors among medical students at King Abdul-Aziz University, Jeddah, Saudi Arabia.

**Methods:**

A cross-sectional survey of 417 medical students (138 males; 279 females) was conducted at King Abdul-Aziz University between January and April 2021. Participants were selected using a multi-stage random sampling method. The survey, which consisted of questions from the 26-item Eating Attitudes Test was distributed online using WhatsApp. Binomial logistic regression (univariate and multivariate) was used to identify the influencing factors on eating attitudes.

**Results:**

Among the 417 medical students, the prevalence of eating disorders was 32.1%. Participants’ average age was 21.65±1.51 years. The BMIs of 54.4% of the students were within normal limits; 14.6 % of them were underweight, 19.2 % were overweight, and 11.8 % were obese. Female medical students (P=0.038, OR=1.62) and pre-clinical stage (P=0.007, OR=1.77) were significantly more likely to be associated with high risk on the EAT-26. A significant association was also found between overweight and obesity in the high-risk students (P=0.016, OR=1.69), but no significant association was found between ED risk and age (OR=0.65). The multivariate logistic regression models showed the most common predictors of EDs among the medical students were female gender and being overweight or obese (OR=2.02, OR=2.10, and OR=2.69, respectively).

**Conclusion:**

Eating disorders are common among medical students. The strongest predictors of eating disorders among the study group were female gender in the pre-clinical stage and being overweight or obese. This study highlights an underrated health problem among Saudi medical students. The implementation of eating disorders preventive program during medical schools that target females may be helpful. Further research is needed to address the best preventive and treatment strategies for EDs among medical students.

## INTRODUCTION

Eating disorders (EDs) are ’behavioural conditions characterized by severe and persistent disturbances in eating behaviours and are associated with distressing thoughts and emotions’.^1^ They are multifactorial, with genetic, biological, psychological, developmental, and sociocultural parameters.^2^ Given their life-threatening complications (e.g. hypoglycaemia) and high morbidity and mortality rates,^3^ EDs have become a major psychiatric and public health issue. Common EDs include bulimia nervosa, binge-eating disorder, and anorexia nervosa, and new EDs, (e.g. orthorexia nervosa) have emerged due to cultural variations.^4^

Anorexia nervosa, which is characterized by anxiety about weight gain, typically leads to below-average weight. Bulimia involves binge eating, covertly at first, followed by purging using unhealthy methods to avoid consuming unwanted calories. However, their causes are not understood.^1^

EDs are prevalent among teens and young adults, and their incidence has increased among medical students.^3^ Young age, academic stressors, workload, and dealing with death and disease are contributing factors to the development of EDs among medical students, with an astonishingly high global prevalence of 10.4%.^5^ Previously considered a Western concern, EDs are now recognized in Asian populations. Approximately 15% of female medical students in the United States have an eating disorder history,^6^ and 31.4% of first- and second-year allied healthcare undergraduates in a Lebanese medical university had EDs.^7^ These disorders are more prevalent in females because of their desire to be thin, dissatisfaction with their physical appearance,^8^ and media pressure to achieve an ideal perfect body.^9^ Preventive measures, such as education, screening, and the early detection of eating disorders^9^,^10^ are needed to address future physicians’ vulnerability to an ailment that could interfere with their medical judgment. Given the insufficient information on eating disorders in Saudi Arabia^9^ and a tendency of studies to focus on young adolescent girls rather than young women and men,^10^ we examined the prevalence of EDs and their associated risk factors among medical students at King Abdul-Aziz University.

## METHODS

This electronic cross-sectional survey was conducted between January and April 2021 at King Abdul-Aziz University in Jeddah, Saudi Arabia, which is a public institution. It was ranked as the #1 Arab university by *Times Higher Education* in 2021.Second- to sixth-year medical students were eligible to participate in the study. Sample size was calculated using Raosoft online sample size calculator. The calculation was based on 95% confidence interval and 5% margin of error. The calculated sample size was 306 participants. Participants were selected using multi-stage random sampling. All of them were informed of the study’s conditions; those who signed the consent form were included and those who declined or failed to complete the survey were excluded. Two randomly chosen representatives from each academic year (second to sixth) distributed the link to the online questionnaire via WhatsApp.

### Data collection

The information collected included participants’ age, gender, academic year, academic stage, and height and weight; the formula weight/[height]^2^ was used to calculate the BMI.^2^

### Eating Attitudes Test (EAT-26)^11^

The survey included questions in English from the 26-item Eating Attitudes Test (EAT-26), which is a questionnaire for screening EDs. Responses to items are rated on a six-point scale ranging from 1 (never) to 6 (always); the total score ranges from zero to 78 and a score ≥20 indicates high risk. The questions elicit information about respondents’ use of diet pills, episodes of excess eating, self-purging, bowel movements, increasing pill or diuretic consumption, and weight loss over the past six months.

### Ethical approval

The Research Ethics Committee of the Faculty of Medicine at King Abdul-Aziz University granted permission to conduct the study (Ref. 584-20, dated 18 November 2020)

### Statistical Analysis

Data were analyzed using SPSS software version 21. Continuous variables are reported as mean±standard deviation, and qualitative variables are reported as frequencies and percentages. Logistic regression was performed to analyze associations, and Pearson’s chi-square and correlation coefficient were used to analyze associations between outcomes and various attributes. P <0.05 was considered significant.

## RESULTS

This study was conducted with 417 medical students with a mean age of 21.65 ±1.51 years (minimum 18, maximum 27); 33.1% were males, 66.9% were females, 74.3% were older than 20 years of age, and the majority were in the clinical academic stage of their medical education. About 19.2% of the students were overweight and 11.8% were obese. The mean height, weight, and body mass index were 1.64 ± 0.08, 63.83 ± 18.83, and 23.47 ± 5.5, respectively. Furthermore, the mean Eating Attitude score was 18.39 ± 14.78. See Table-I for socio-demographic and anthropometric characteristics of participants are given in Tale-I.

**Table-I:** Socio-demographic and anthropometric characteristics of the participants.

Characteristic	Participants (N=417)	

	N	%
***Gender***		
Males	138	33.1
Females	279	66.9
***Age (years)***		
Less than 20	107	25.7
More than 20	310	74.3
***Academic year***		
2^nd^ year	81	19.4
3^rd^ year	106	25.4
4^th^ year	77	18.5
5^th^ year	79	18.9
6^th^ year	74	17.7
***Academic stage***		
Pre-clinical	187	44.8
Clinical	230	55.2
***BMI***		
Within normal limits	227	54.4
Underweight	61	14.6
Overweight	80	19.2
Obese	49	11.8
Characteristic	Participants (N=417) Mean±SD	
Age (years)	21.65±1.51	
Weight (kg)	63.82±18.83	
Height (metre)	1.64±0.08	
BMI (kg/m^2^)	23.47±5.5	
EAT-26 mean score	18.39±14.78	

BMI, body mass index;

EAT-26, 26-item Eating Attitudes Test.

The outcome was defined as having a score more than 20 on the EAT-26 (20 was the cut-off score). A total of 283 (67.9%) students scored ≤20 points on the EAT-26 and 134 (32.1%) scored >20, indicating an ED prevalence of 32.1% (Table-II).

**Table-II:** Prevalence of eating disorders among medical students at King Abdulaziz University Using EAT-26 Questionnaire.

Eating Attitudes Test-26 (EAT-26)	Frequency	Percent
Low Risk (less tdan or equal to score 20)	283	67.9
High Risk (more tdan score 20)	134	32.1

Female medical students (P=0.038, OR=1.62) and pre-clinical stage (P=0.007, OR=1.77) were significantly more likely to be associated with high risk on the EAT-26 (Table-III). A significant association was also found between overweight and obesity in the high-risk students (P=0.016, OR=1.69), but no significant association was found between ED risk and age (P=0.068, OR=0.65). Correlation analysis showed that 68.7% of medical students older than 20 years fell in the high-risk category (r=-0.139, P=0.005). Those with a BMI indicating overweight or obesity were identified as ‘high risk’ on the EAT-26 [*n*=52 (38.8%), r=0.133, OR=1.69].

**Table-III:** Relationship between eating-disorder risk and participants’ socio-demographic and anthropometric characteristics (univariate regression analysis).

Sociodemographic Characteristic	Participants’ Risk of Developing an Eating Disorder (N=417)		P-value	OR (95% CI)

	High risk (n=134) n (%)	Low risk (n=283) n (%)		
***Gender***				
Males	35 (26.1)	103 (36.4)	0.038^*^	1.62
Females	99 (73.9)	180 (63.6)		(1.02-2.55)
***Age (years)***				
Less than 20	42 (31.3)	65 (23.0)	0.068	0.65
More than 20	92 (68.7)	218 (77.0)		(0.41-1.03)
***Academic stage***				
Pre-clinical	73 (54.5)	114 (40.3)	0.007*	1.77
Clinical	61 (45.5)	169 (59.7)		(1.16-2.68)
***BMI***				
Normal and underweight	82 (61.2)	206 (72.8)	0.016^*^	1.69
Overweight and obese	52 (38.8)	77 (27.2)		(1.09-2.62)

OR, odds ratio; CI, confidence interval; BMI, body mass index

The multivariate logistic regression models showed the most common predictors of EDs among the medical students were female gender and being overweight or obese (Table-IV). The crucial roles of gender and BMI in the mechanisms underlying EDs remain unclear. These roles are supported by the P-value of 0.007 for gender, 0.009 for overweight, and 0.004 for obesity, and the adjusted ORs of 2.02 for gender and 2.69 for obesity. However, no relationship was found between age and atypical eating habits (Table-IV).

**Table-IV:** Predictors of eating disorders among the participants (multivariate regression analysis).

Characteristic	P-value	AOR	95% CI	

			Lower	Upper
***Gender***				
Female	0.007*	2.02	1.20	3.38
Age (years)				
More tdan 20	0.349	0.75	0.42	1.35
***Academic stage***				
Clinical	0.098	0.64	0.37	1.08
BMI				
***Underweight***	0.920	0.96	0.51	1.82
Overweight	0.009*	2.10	1.19	3.71
Obese	0.004*	2.69	1.35	5.35

AOR, adjusted odds ratio; CI, confidence interval; BMI, body mass index; * significant.

Analyses of eating and exercise over the past six months among high-and low-risk students showed that all of the behaviours associated with EDs were significantly more prevalent in the high-risk group (Table-V). Binge-eating episodes were significantly more frequent among high-risk participants, with 19.4% binge eating two to three times/month compared to only 9.8% in the low-risk group. Furthermore, 8.2% of the high-risk students binged two to three times/week compared to 4.9% in the low-risk group; likewise, 6.7% of students in the high-risk group self-induced vomiting two to three times/month, and 1.5% vomited two to six times/week compared to the lower rates among the low-risk students. The use of laxatives, diet pills, and diuretics, and exercising longer than sixty minutes/day were more common among high-risk students; 17.2% of them exercised at least two to six times/week and 10.4% exercised at least once daily. Weight loss >9 kg within the past six months was reported by 76.1% of the high-risk students and 91.2% of the low-risk students.

**Table-V:** Frequencies of behaviors related to eating disorder risk, as reported by participants on the EAT-26.

Behaviour-related Questions 1-4 In the past six months how often have you	Eating Disorder Risk among Study Participants (N=417)		Chi-square (P-value)

	High risk (n=134) n (%)	Low risk (n=283) n (%)	
1. Gone on eating binges in which you felt you might not be able to stop?			
Never	29 (21.6)	154 (54.4)	
Once a montd or less	45 (33.6)	52(18.4)	
2-3 times a montd	26 (19.4)	28 (9.9)	40.84 (<0.001)
Once a week	13 (9.7)	21 (7.5)	
2-6 times a week	11 (8.2)	14 (4.9)	
Once a day or more	10 (7.5)	14 (4.9)	
2. Made yourself sick (vomited) to control your weight or shape?			
Never	2 (68.7)	262 (92.6)	
Once a montd or less	24 (17.9)	4 (1.4)	
2-3 times a montd	9 (6.7)	7 (2.5)	51.43 (<0.001)
Once a week	1 (0.7)	0 (0.0)	
2-6 times a week	2 (1.5)	1(0.4)	
Once a day or more	6 (4.5)	9 (3.2)	
3. Used laxatives, diet pills, or diuretics to control your weight or shape?			
Never	91 (67.9)	255 (90.1)	
Once a montd or less	16 (11.9)	13 (4.6)	37.58 (<0.001)
2-3 times a montd	11 (8.2)	4 (1.4)	
Once a week	5 (3.7)	1 (0.4)	
2-6 times a week	4 (3.0)	1 (0.4)	
Once a day or more	7 (5.2)	9 (3.2	
4. Exercised longer tdan 60 minutes a day to lose or to control your weight?			
Never	39 (29.1)	157 (55.5)	
Once a montd or less	21 (15.7)	43 (15.2)	
2-3 times a montd	17 (12.7)	20 (7.1)	31.32 (<0.001)
Once a week	20 (14.9)	24 (8.5)	
2-6 times a week	23 (17.2)	29 (10.2)	
Once a day or more	14 (10.4)	10 (3.5)	
5. Have you lost 20 pounds (9 kg) or more in the past six months?			
Yes	102 (76.1)	258 (91.2)	17.44 (<0.001)
No	32 (23.9)	25 (8.8)	

## DISCUSSION

The prevalence of unhealthy dietary habits, as measured by EAT-26 scores, among the medical students in our study was high, with 32.1% reporting high-risk behaviours, compared to 22.8% of medical students in a study conducted in Karachi,^12^ and 17%, of students in a Lebanese medical school.^13^ We reported more high-risk behaviours than did other studies with medical students, and our findings were similar to the results of studies with non-clinical university students. For example, 35.4% of female university undergraduates from four colleges in Saudi Arabia were found to have a high-risk for EDs,^9^ and 29.4% of females in a university preparatory program in Saudi Arabia were classified ‘at a high level of concern’ for developing an ED.^10^ Yet a prevalence of high-risk behaviors was found in only 9.3% of Latino college freshmen students.^14^ Hence, differences in prevalence rates of EDs are difficult to interpret, mostly because of an insufficient number of studies.^14^

Females are more likely to show a tendency toward EDs than males are,^15^ and they are more likely to diet to control their weight and to have low self-esteem, which might increase their likelihood of developing EDs.^16^-^18^ Female and male university students in northwest Pakistan reported different risk factors for EDs; they only had ‘poor sleep’ in common as a risk factor.^15^ Females have a greater propensity than males for developing bulimia,^19^ and in our study, binge-eating episodes were significantly more frequent among the high-risk participants, with 19.4% of students binge eating two to three times/month compared to only 9.8% in the low-risk group.

The medical students in our study with a BMI of 25.0–29.9 kg/metre^2^ or ≥30.0 kg/metre^2^ reported high-risk dietary behaviors more often than did students with a lower BMI. Individuals with a normal BMI tend to use healthy dietary habits and exercise to maintain their weight, which explains these findings.^12^

Anorexia and bulimia have multiple medical complications, consistent with the degree of weight loss or the mode and frequency of purging.^20^ Without treatment, they can result in severe disability or death. Long-term consequences that develop during adolescence include adverse reproductive-related outcomes (e.g. preterm births and miscarriage) among other health consequences.^21^ However, early detection and treatment by physicians can significantly improve outcomes and reduce morbidity;^20^ in some cases, they may even motivate medical students to become better doctors.

Given the association of eating disorders with mental illness, medical students, like many doctors, sometimes wait until a crisis develops to seek medical help, in order to avoid stigmatisation.^22^ They often neglect their symptoms and self-medicate instead, which conceals psychological or psychiatric issues they might have.^23^ Education (awareness) is the first path to reducing self- and other-stigmatisation.

### Limitations

First, its cross-sectional design precludes causal inferences. Second, the study setting was located in a single city, consisting of an urban population; therefore, generalization of the findings cannot be made. We suggest further studies involving multi-cultural and more heterogeneous populations with a focus on rural populations.

## CONCLUSION

Based on our results, EDs are common among medical students. The strongest predictors of eating disorders among the study group were female gender in the pre-clinical stage and being overweight or obese. This study highlights an underrated health problem among Saudi medical students. Raising awareness and early detection of EDs among medical students are important. The implementation of eating disorders preventive program during medical schools that target females may be helpful. Healthy eating habits should be addressed by trained specialist at an institutional level. Further research is needed to address the best preventive and treatment strategies for EDs among medical students.

### Authors’ Contribution:

**RG:** Study design, statistical analysis, manuscript writing, revision, responsible for accuracy and integrity of work.

**AA, LA, SA:** Study design, data collection and manuscript revision.

**MB:** Study design, data collection and manuscript revision.
